# Double Dosing Levonorgestrel-Based Emergency Contraception for Individuals With Obesity: A Randomized Controlled Trial

**DOI:** 10.1097/AOG.0000000000004717

**Published:** 2022-06-07

**Authors:** Alison B. Edelman, Jon D. Hennebold, Kise Bond, Jeong Y. Lim, Ganesh Cherala, David F. Archer, Jeffrey T. Jensen

**Affiliations:** Department of Obstetrics & Gynecology and the Biostatistics Shared Resource, Knight Cancer Institute, Oregon Health & Science University, Portland, and the Division of Reproductive & Developmental Sciences, Oregon Primate National Research Center, Beaverton, Oregon; Clinical Pharmacology, Gilead Sciences, Foster City, California; and the Clinical Research Center, Department of Obstetrics and Gynecology, Eastern Virginia Medical School, Norfolk, Virginia.

## Abstract

**OBJECTIVE:**

To assess whether dose escalation (ie, doubling the dose) of emergency contraception that contains levonorgestrel (LNG) improves pharmacodynamic outcomes in individuals with obesity.

**METHODS:**

We enrolled healthy, reproductive-age individuals with regular menstrual cycles, body mass index (BMI) higher than 30, and weight at least 176 lbs in a randomized pharmacodynamic study. After confirming ovulation (luteal progesterone level greater than 3 ng/mL), we monitored participants with transvaginal ultrasonography and blood sampling for progesterone, luteinizing hormone, and estradiol every other day until a dominant follicle measuring 15 mm or greater was visualized. At that point, participants received either oral emergency contraception with LNG 1.5 mg or 3 mg (double dose) and returned for daily monitoring for up to 7 days. Our primary outcome was the difference in the proportion of participants with no follicle rupture 5 days postdosing (yes or no) between groups. The study had 80% power to detect a 30% difference in the proportion of cycles with at least a 5-day delay in follicle rupture (50% decrease).

**RESULTS:**

A total of 70 enrolled and completed study procedures. The two groups had similar baseline demographics (mean age 28 years, BMI 38). We found no difference between groups in the proportion of participants without follicle rupture more than 5 days post–LNG dosing (LNG 1.5 mg: 18/35 [51.4%]; LNG 3.0 mg: 24/35 [68.6%], *P*=.14). Among participants with follicle rupture before 5 days, the time to rupture did not differ between groups (day at 75% probability of no rupture is day 2 for both groups).

**CONCLUSION:**

Individuals with higher BMIs and weights experience a higher risk of failure of emergency contraception with LNG and exhibit an altered pharmacokinetic profile. However, the simple strategy of doubling the dose does not appear to be an effective intervention to improve outcomes.

**CLINICAL TRIAL REGISTRATION:**

ClinicalTrials.gov, 02859337.

## Abstract 2

Increasing the dose of levonorgestrel-containing emergency contraception from 1.5 mg to 3 mg did not improve rates of ovulation delay in individuals with obesity.

Emergency contraception provides an individual with an additional line of defense against unintended pregnancy after an act of unprotected intercourse. The orally dosed emergency contraceptives levonorgestrel (LNG) and ulipristal acetate work by inhibiting or delaying ovulation, respectively, and reduce the risk of pregnancy for a single act of unprotected intercourse by up to 90%.^1–4^ Emergency contraception containing LNG is widely accessible and available in many countries over-the-counter without a prescription.

Unfortunately, obesity is a known risk factor for the failure of emergency contraception that contains LNG, which is a significant problem given the current global obesity epidemic.^5–7^ Glasier et al^5^ reanalyzed data from two large randomized control trials to identify risk factors for emergency contraception failure. Individuals with body mass indexes (BMIs, calculated as weight in kilograms divided by height in meters squared) in the obese range (higher than 30) who used emergency contraception containing LNG had more than a fourfold greater risk of pregnancy compared with individuals with BMIs in the normal range (odds ratio 4.41, 95% CI 2.05–9.44); individuals with BMIs in the overweight range (25–29.9) were twice as likely to experience failure. This reanalysis also found failure associated with high body weight; emergency contraception that contains LNG appears to have a ceiling of efficacy at 70 kg and no emergency contraception efficacy for those weighing 80 kg or more.

As a single-dose therapy, emergency contraception is reliant on achieving a rapid peak drug level at a critical time point before the luteinizing hormone (LH) surge.^8–10^ We hypothesized that individuals with obesity did not achieve an LNG concentration sufficient to block the LH surge after oral dosing. Prior research from our group and others demonstrated that key pharmacokinetic parameters for emergency contraception with LNG (1.5 mg) were 50% lower in individuals with obesity, as compared with those with BMIs in the normal range.^11,12^ We then tested a dose escalation strategy (3 mg of emergency contraception with LNG) in an effort to counteract obesity-related pharmacokinetic changes and successfully reestablished a normal LNG pharmacokinetic profile in individuals with obesity.^12^ Although some practice recommendations, including the Faculty of Sexual and Reproductive Healthcare’s 2017 emergency contraception guidelines,^13^ moved forward to recommend this “take two” strategy, we lack clinical data with improved pharmacodynamic (pharmacodynamic) outcomes to support this recommendation.^14^ To further evaluate the potential of double dosing as a management strategy for individuals with higher BMIs and weights, we designed a study to determine the effects of 3 mg of LNG on preventing ovulation.

## METHODS

We conducted a randomized controlled trial at Oregon Health & Science University in Portland, Oregon, and Eastern Virginia Medical School in Norfolk, Virginia, from June 2017 to February 2021. The Institutional Review Boards at Oregon Health & Science University and Eastern Virginia Medical School approved the study protocol. We recruited healthy individuals aged 18–35 years with regular menstrual cycles (21–35 days) and BMIs higher than 30 with weights of 176 lbs or more. Participants were compensated for their time. Individuals were required not to be at risk for pregnancy (abstinent or using a nonhormonal method of contraception, eg, pills, patches or rings; injection; or hormonal intrauterine device). Major exclusion criteria obtained through patient report, review of medical records, and clinical exam included: metabolic disorders such as uncontrolled thyroid dysfunction and polycystic ovarian syndrome or clinical evidence of androgen excess; impaired liver or renal function; actively seeking or involved in a weight loss program (must be weight stable); pregnancy, breastfeeding, or seeking pregnancy; recent (8 weeks) use of hormonal contraception; current use of drugs that interfere with metabolism of sex steroids; smoking or vaping; and chronic marijuana use.

Individuals underwent an initial phone screen and then, if eligible, completed an in-person screening visit to collect baseline demographics including race–ethnicity per National Institutes of Health reporting guidelines, health information, and a serum progesterone level during luteal phase based on an individual's cycle length to confirm ovulatory status (progesterone level at 3 ng/mL or more), an inclusion criterion for participation. All participants completed written informed consent before any study procedures. Participants started their treatment cycle at the time of next menses or could delay for 1–2 menstrual cycles if a scheduling conflict occurred. On days 6–8 of the treatment cycle, we began monitoring participants every other day for follicular activity with transvaginal ultrasonographic examination, as well as blood sampling for progesterone, estradiol, and LH until a dominant follicle measuring 15 mm or greater in at least one dimension was visualized.^15,16^ After confirming a negative pregnancy test, we randomized individuals to a 1.5 mg or 3 mg dose of LNG, and study staff directly observed ingestion of the drug. The Oregon Health & Science University research pharmacy maintained the computer-generated randomization scheme and kept treatment assignments in a locked database. Postdosing, we monitored participants daily with transvaginal ultrasonography and blood sampling until evidence of follicle rupture (more than 50% reduction of mean size or complete disappearance of follicle) or for up to a total of 7 days if no evidence of rupture occurred.^4,15,16^

We hypothesized that doubling the LNG dose would result in a higher proportion having ovulation prevented and with the clinical expectation of greater efficacy as emergency contraception. Our primary outcome was the difference in the proportion of participants with no follicle rupture of 5 days postdosing (yes or no) between groups. This outcome is based on the length of time sperm are believed to remain viable in the female genital tract for 5 days.^4,17^ Our main secondary outcome compared the timing of follicle rupture between groups. If the date of follicle rupture was unclear by ultrasonographic imaging (eg, collapse was seen, but reduction of size was less than 50%), we used serum hormone levels to adjudicate day of ruptures. Two investigators independently reviewed these cycles while being masked to treatment allocation; if disagreement occurred, a third investigator was used. An investigator masked to treatment allocation and ultrasonographic data also identified any cycles based on hormone data where drug dosing may have occurred too late, after LH rise, when treatment effects of LNG might have waned partially or completely. The study had an 80% power to determine a 30% difference, with a 5% significance level, in the proportion of cycles with at least a 5-day delay in follicle rupture (yes or no) with a sample size of 62. We planned to enroll 70 women to account for drop outs. We planned to perform our analyses using intent-to-treat as well as per protocol (excluding cycles where LNG dose timing was suboptimal). We used the REDCap electronic capture tool at Oregon Health & Science University for data management and exported data directly from REDCap into SAS 9.4 for statistical analysis. We summarized demographics and baseline clinical characteristics using descriptive statistics such as mean (SD) and count (%) and compared between the two randomized arms to ensure equality. The primary outcome was analyzed with the χ^2^ test. The secondary outcome, which is a time to rupture, was assessed using Kaplan-Meier and log-rank tests. Time to rupture was censored at day 5.

Hormone assays were performed at the Endocrine Technologies Support Core at the Oregon National Primate Research Center (http://www.ohsu.edu/xd/research/centers-institutes/onprc/research-services/research-support/endocrine-technology.cfm). The Endocrine Technologies Support Core uses a Roche Cobas e411 chemiluminescence-based automatic clinical platform for analyses of serum estradiol, progesterone, and LH, with sensitivities of 5 pg/mL, 30 pg/mL, and 0.1 milli-international units/mL, respectively. The intra-assay and interassay variation with the Roche e411 is consistently less than 7% for all assays. Quality control samples and validations were repeated before each assay run.

## RESULTS

**Fig. 1.**
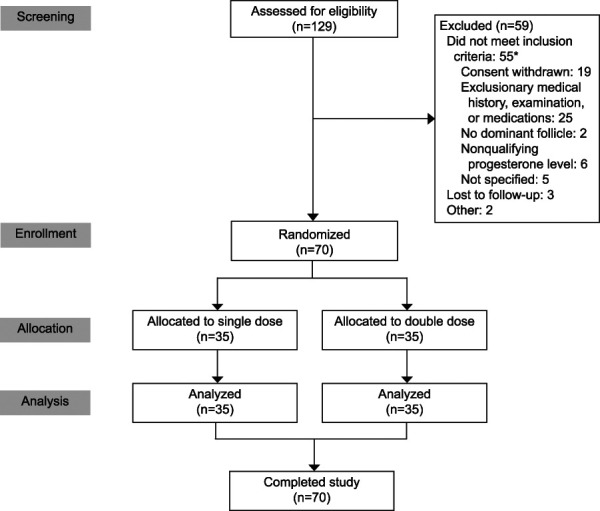
CONSORT (Consolidated Standards Reporting Trials) flow diagram. *Items not mutually exclusive. Edelman. Emergency Contraception and BMI. Obstet Gynecol 2022.

A total of 70 women enrolled and completed all study procedures (35 per study arm, Fig. 1). The two groups had similar baseline demographics (Table 1); mean age was 28 years, mean BMI was 38, most identified as White not Hispanic, and about half reported never experiencing a pregnancy. At the time of LNG dosing, the average largest follicle measurement was similar in both groups (LNG 1.5 mg: 16.3 mm [SD 1.7]; LNG 3.0 mg: 15.9 mm [SD 1.1]). We identified four individuals who required adjudication to determine the date of follicle rupture (two participants from each group). Follicle collapse occurred for these four individuals, but the collapse did not achieve a 50% decrease in size. Two participants maintained their classification as no rupture before 5 days (one participant from each group), and two participants were reclassified as ruptured before 5 days (one participant from each group). This reclassification had no effect on the results of our primary or secondary outcomes.

**Table 1.**
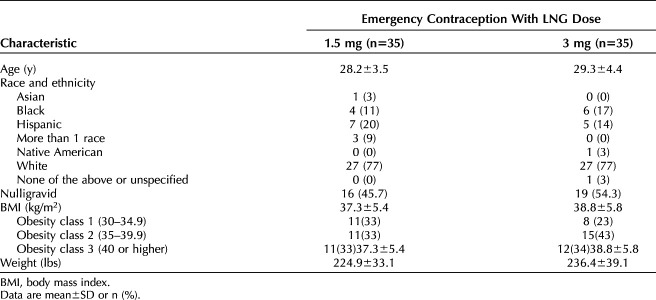
Characteristics of Study Participants

**Fig. 2.**
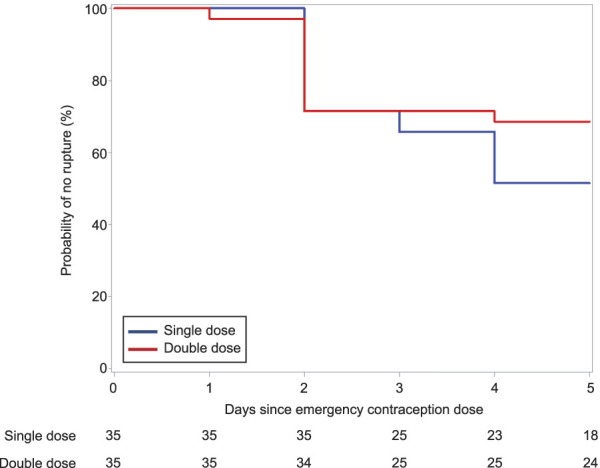
Kaplan-Meier curves for time to rupture after levonorgestrel (LNG) 1.5 mg (*blue line*) and LNG 3.0 mg (*red line*) at time zero. The study groups were no different in their time to follicle rupture (log-rank test *P*=.2). Time to rupture was censored at day 5. Edelman. Emergency Contraception and BMI. Obstet Gynecol 2022.

We found no difference between groups in the intention-to-treat analysis of our primary outcome, the proportion of participants who achieved at least 5 days without evidence of follicle rupture post–LNG dosing. Approximately half of individuals in both groups achieved 5 days or more with no evidence of rupture (LNG 1.5 mg: 18/35 [51.4%]; LNG 3.0 mg: 24/35 [68.6%], *P*=.14). Our secondary outcome, time to follicle rupture before 5 days, was also no different between groups (day at 75% probability of no rupture is day 2 for both groups, *P*= .21, Fig. 2). Additionally, we performed a per protocol analysis excluding 9 individuals (LNG 1.5 mg: 6; LNG 3.0 mg: 3) where drug dosing may have occurred too late, after LH rise. We still found no differences between groups for either our primary outcome (LNG 1.5 mg: 17/29 [58.6%]; LNG 3.0 mg: 23/32 [71.9%], *P*=.28) or the time to rupture (day at 75% probability of no rupture is day 3 for both groups, *P*=.36).

We further analyzed the 27 participants that experienced follicle rupture before 5 days to identify whether rupture was preceded by any LH rise or a LH rise of at least 21 international units/L. All but one participant in the LNG 3.0 mg group experienced an LH rise preceding follicle rupture, with no differences found between LNG dosing groups. A similar proportion in each group experienced a LH rise of at least 21 international units/L (LNG 1.5 mg: 7/17, 41%; LNG 3.0 mg 8/11, 72%, *P* 0.14).

## DISCUSSION

We designed this study to assess whether a higher dose of emergency contraception with LNG, 3 mg, could improve the rate of ovulation inhibition when given in the periovulatory interval to individuals with higher BMIs and weights in comparison with the standard 1.5-mg dose. We found no differences between LNG dosing groups in the proportion of individuals with no follicle rupture for at least 5 days. We also found no differences in the time to follicle rupture before 5 days. Although we have previously shown that dose escalation corrects the observed pharmacokinetic differences with LNG dosing in individuals with obesity, this strategy did not translate to improved end-organ results as assessed by ovulation.^18,19^ Our findings have important clinical relevance for individuals BMIs in the obese range needing emergency contraception. Our results do not support clinical recommendations for double dosing of emergency contraception with LNG in individuals with BMIs higher than 30 and weights of at least 176 lbs.

Emergency contraception with LNG works by preventing the LH surge, blocking follicle rupture. To block the hypothalamic-pituitary axis and ovulation, a progestogen must circulate at a concentration that is above the ovulatory inhibitory level. For contraceptives with LNG, the ovulatory inhibitory concentration is considered 0.2 pg/mL.^20^ Our prior pharmacokinetic studies have demonstrated that, compared with women with normal BMIs, women with BMIs in the obese range experience an approximately 50% reduction in plasma exposures of LNG.^18^ Because the success of oral emergency contraception depends on reaching an inhibitory concentration before the onset of the LH surge, significantly lower LNG exposures could influence efficacy. Therefore, we hypothesized that pharmacokinetic alterations could explain the difference in outcomes with emergency contraception with LNG observed in clinical efficacy trials. Our subsequent results, which demonstrated that a 3-mg dose of LNG corrected the observed pharmacokinetic abnormalities, supported further evaluations.^18^ Results from this detailed pharmacodynamic study of double dosing suggest that pharmacokinetic differences do not explain the clinical differences in efficacy. The increased dose of LNG did not reduce the risk or timing of ovulation among individuals with obesity who received the dose at an optimal time in the cycle, before the LH surge.

Free (unbound) drug is often considered to be pharmacologically relevant. However, for endogenous and exogenous agents (ie, LNG), the fraction of drug that is bound to sex hormone binding globulin is considered bioactive. Levonorgestrel is extensively (approximately 98%) plasma protein bound, of which approximately 65% is bound to sex hormone binding globulin.^21^ Hence, it could be postulated that normalization of sex hormone binding globulin bound LNG is the more critical component than simply normalizing plasma concentrations of LNG. Sex hormone binding globulin levels are lower in those with BMIs in the obese range. Earlier, we demonstrated normalization of free levels of LNG on double dosing^18^; however, the distribution of bound forms between albumin (pharmacologically inactive) and sex hormone binding globulin (bioactive) is unclear. Alternatively, or in addition to differences in albumin–sex hormone binding globulin binding, obesity-related differences may occur with hypothalamic-pituitary-ovarian responsiveness to LNG.^22,23^ LNG dose optimization in conjunction with normalization of sex hormone binding globulin bound LNG may be needed to achieve improved efficacy.

Strengths of our study include the randomized design with independent groups and rigorous assessment of outcomes. Although a crossover design allows each participant to serve as her own control, we have found it difficult to retain participants willing to undergo repeated intensive interventions over multiple cycles (baseline, treatment, washout, treatment). Our study design was based on an indirect marker of pregnancy, ovulation. However, pregnancy does not occur without ovulation. We based our study design on the methodology used in other emergency contraception comparator studies.^2,4,15,16^ We did adjudicate several cycles to determine the date of follicle rupture based on hormone data, but this process had no effect on our main outcomes. Retrospectively, we identified potential cycles where LNG was administered too late to be optimally effective or ineffective (too close to the LH rise or surge). These per protocol analyses also found no differences between groups.

Our study includes the following limitations. First, we used follicle rupture as a surrogate for ovulation. Evaluation of clinical pregnancy rate, the true outcome of interest, is not feasible or ethical in a pharmacodynamic study. We confirmed ovulatory status before study enrollment with elevated progesterone during the presumptive luteal phase, but we did not have participants undergo a baseline cycle with ultrasonography and hormone monitoring. Thus, some included individuals may have unrecognized baseline ovulatory dysfunction, but these individuals should have been randomly distributed between groups. We also did not evaluate the normalcy of the luteal phase during treatment cycles, because we stopped monitoring individuals as soon as rupture occurred. It is possible that abnormal ovulations and luteal insufficiency could create a difference in pregnancy rates. Prior pharmacodynamic studies of emergency contraception with LNG do combine the categories of “dysfunctional” and no ovulation to demonstrate emergency contraception effectiveness, but the contribution of “dysfunction” to the effectiveness of the method is unclear. Further insight regarding pregnancy rates in individuals with BMIs of 30 or higher after 3 mg LNG are forthcoming; as a phase IIb emergency contraception study recently closed to enrollment (NCT03537768).^4^ Finally, we did not enroll an additional comparison group of women with normal BMIs. However, a prior study has shown ovulation rates similar to those seen in our study with standard emergency contraception dosing in individuals with normal BMIs.^4^

Our results do not support a recommendation of double dosing of LNG for emergency contraception in individuals with obesity. Clinical studies suggest that emergency contraception with LNG does not work in women who weigh more than 70 kg, but that ulipristal acetate appears effective. Further research evaluating the mechanisms of failure for hormonal emergency contraception in those with obesity may provide additional insights. Until then, use of ulipristal acetate for emergency contraception in individuals with obesity who desire an oral agent seems likely to provide the best outcomes.

**Authors' Data Sharing Statement**

Will individual participant data be available (including data dictionaries)? *No*.
What data in particular will be shared? *Not available*.
What other documents will be available? *Not available*.
When will data be available (start and end dates)? *Not applicable*.
By what access criteria will data be shared (including with whom, for what types of analyses, and by what mechanism)? *Oregon Health & Science University understands and agrees to comply with the NIH policy on Sharing Research Data.*
